# Dorsal raphe serotonergic neurons promote arousal from isoflurane anesthesia

**DOI:** 10.1111/cns.13656

**Published:** 2021-05-11

**Authors:** Ao Li, Rui Li, Pengrong Ouyang, Huihui Li, Sa Wang, Xinxin Zhang, Dan Wang, Mingzi Ran, Guangchao Zhao, Qianzi Yang, Zhenghua Zhu, Hailong Dong, Haopeng Zhang

**Affiliations:** ^1^ Department of Anesthesiology and Perioperative Medicine Xijing Hospital Fourth Military Medical University Xi’an China

**Keywords:** dorsal raphe, general anesthesia, isoflurane, serotonin

## Abstract

**Aims:**

General anesthesia has been widely applied in surgical or nonsurgical medical procedures, but the mechanism behind remains elusive. Because of shared neural circuits of sleep and anesthesia, whether serotonergic system, which is highly implicated in modulation of sleep and wakefulness, regulates general anesthesia as well is worth investigating.

**Methods:**

Immunostaining and fiber photometry were used to assess the neuronal activities. Electroencephalography spectra and burst‐suppression ratio (BSR) were used to measure anesthetic depth and loss or recovery of righting reflex to indicate the induction or emergence time of general anesthesia. Regulation of serotonergic system was achieved through optogenetic, chemogenetic, or pharmacological methods.

**Results:**

We found that both Fos expression and calcium activity were significantly decreased during general anesthesia. Activation of 5‐HT neurons in the dorsal raphe nucleus (DRN) decreased the depth of anesthesia and facilitated the emergence from anesthesia, and inhibition deepened the anesthesia and prolonged the emergence time. Furthermore, agonism or antagonism of 5‐HT 1A or 2C receptors mimicked the effect of manipulating DRN serotonergic neurons.

**Conclusion:**

Our results demonstrate that 5‐HT neurons in the DRN play a regulative role of general anesthesia, and activation of serotonergic neurons could facilitate emergence from general anesthesia partly through 5‐HT 1A and 2C receptors.

## Graphical abstract

General anesthesia decreases the activities of serotonergic neurons in the dorsal raphe. Activation of DRN 5‐HT neurons reduced the depth of anesthesia and accelerated the emergence from general anesthesia, partially through the 5‐HT 1A and 2C receptors.

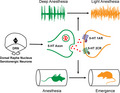

## 1 INTRODUCTION

General anesthesia, with the ability to cause reversible loss and recovery of consciousness, has been widely applied in surgical or multiple diagnostic medical procedures, but the mechanisms behind remain elusive.^1^, ^2^, ^3^, ^4^ Because of the similarity of the brain states during general anesthesia and non‐rapid eye movement (NREM) sleep,^5^, ^6^ several neuromodulators involving in sleep regulation have been shown to play a role in general anesthesia, including dopamine, norepinephrine, acetylcholine, and hypocretin.^7^, ^8^, ^9^, ^10^, ^11^ Along with its broad participation in regulating multiple brain functions like depression and cognition, 5‐hydroxytryptamine (5‐HT, also known as serotonin) has long been implicated in the regulation of sleep and wakefulness.^12^ In fact, several studies have reported that 5‐HT levels were fluctuating during anesthesia, indicating a regulatory role of 5‐HT system in general anesthesia.^13^, ^14^ However, how 5‐HT and serotonergic neurons regulate general anesthesia has been poorly studied.

Serotonergic (5‐HTergic) neurons in the dorsal raphe nucleus (DRN) provide dominant projections to the midbrain and forebrain and are innervated by cerebral cortex, limbic systems, basal forebrain, and hypothalamus.^15^, ^16^, ^17^ As a component of ascending arousal systems, studies have shown that activation of 5‐HT neurons by optogenetics significantly prolonged the wakefulness and decreased the NREM sleep.^18^ Recently, an elaborately performed study demonstrated the bidirectional role of serotonergic system in the maintenance of sleep homeostasis and the regulation of sleep/wake cycle.^19^ We previously reported that orexin facilitated emergence from isoflurane anesthesia partially through activating 5‐HT neurons in the DRN, but there is no direct evidence supporting the casual effect of DRN 5‐HT neurons themselves on the effect of general anesthesia.^20^

In the present study, we performed the immunostaining and calcium indicators combined with fiber photometry to measure the 5‐HT neuronal activity in vitro and in vivo separately. Then, optogenetics and chemogenetics were used to manipulate the DRN 5‐HT neurons and illustrate the effect of 5‐HT neurons on electroencephalographic or behavioral features of isoflurane anesthesia. Finally, the specific subtype of receptors through which serotonin exerted their effect was identified by pharmacological methods.

## 2 METHODS

### 2.1 Animals

All experiments were performed in accordance with the protocols approved by the Ethics Committee for Animal Experimentation and strictly followed the Guidelines for Animal Experimentation of Fourth Military Medical University (Xi'an, China). Sprague Dawley (SD) rats were purchased from Vital River Laboratory Animal Technology Co. Ltd (Beijing, China). Sert‐Cre mice were generously given by Prof. Minmin Luo (National Institute of Biological Sciences, Beijing, China). Rats (2‐3 animals per cage) and mice (3‐5 animals per cage) were housed separately in cages before experiments in a specific‐pathogen‐free environment with a constant temperature of 23°C (22‐24°C) and humidity of 40% (38%‐42%) on a 12/12‐h light‐dark cycle. All animals were provided with food and water ad libitum.

### 2.2 Virus and drugs

Viruses of rAAV2/9‐EF1a‐DIO‐mCherry or rAAV2/9‐EF1a‐DIO‐ChR2‐mCherry were used for optogenetic experiments, rAAV2/9‐EF1a‐DIO‐hM3Dq‐mCherry or rAAV2/9‐EF1a‐DIO‐hM4Di‐mCherry were used for chemogenetic experiments, and rAAV2/9‐EF1a‐DIO‐GCaMP6 s were constructed for in vivo fiber photometry. All aforementioned viruses were provided by BrainVTA Technology Co. Ltd. (Wuhan, China). Isoflurane (Aerrane) for anesthesia was purchased from Baxter Healthcare Corporation (USA). For chemogenetic experiments, Clozapine N‐Oxide (CNO, 3 mg/kg in saline, Cayman Chemical, USA) or vehicle was injected intraperitoneally before the animal was anesthetized. For pharmacological experiments, 5‐HT (H9523), 8‐OH DPAT (H8520), WAY100635 (W108), CP93129 (PZ0102), SB224289 (S201), DOI (D8308), M100907 (M3324), RS102221 (R1658), m‐CPBG (C144), MDL 72222 (T102), SB 399885 (SML0604), LP44 (L9793), and SB269970 (S7389) were purchased from Sigma‐Aldrich (USA), and WAY 208466 (633304‐27‐5) was purchased from Tractus Company (China).

### 2.3 Stereotaxic surgery

Mice were induced to anesthetic states in an induction chamber with 1.2% isoflurane (Baxter Healthcare, Puerto Rico) vaporized by oxygen of 1.0 L/min and then transferred and fixed to a stereotaxic frame while keeping anesthetized by 0.8% isoflurane through a mask. Skull was exposed and cleaned by 10% hydrogen peroxide. Then, viruses were injected into DRN (AP: −4.55 mm, ML: −0.00 mm, DV: −2.55 mm) by Nanoject III (Drummond Scientific, Broomall, PA) injector at a rate of 23 nl/min. For optogenetic and fiber photometry experiments, an optical fiber (diameter: 200 μm, Inper, Hangzhou, China) was implanted to the DRN (AP: −4.55 mm, ML: −0.00 mm, DV: −2.50 mm, 15‐degree angle to vertical axis). Experiments were performed at least 3 weeks after surgery to ensure the expression of viruses. For drug treatments, SD male rats were anesthetized and fixed to the stereotaxic apparatus, after which a guide cannula (RWD, Inc., Shenzhen, China) was stereotaxically directed to the lateral cerebral ventricle (AP: −1.00 mm, ML: −1.90 mm, DV: −2.70 mm). Rats were allowed to recover for at least 7 days after the surgery.

### 2.4 Fiber photometry

For fiber photometry experiments, the implanted optical fiber was attached to the fluorescence photometer (Thinker Tech, Nanjing, China). The beam of 488 nm continuous LED light was emitted and reflected by a dichromic mirror, and GCaMP6 s fluorescence was focused and recorded by the photometer. To reduce the bleaching of the calcium indicator, laser intensity at the fiber tip was kept between 30 and 40 µW. Average ΔF/F values were calculated by a custom written MATLAB code, as (F_duration_ – F_baseline_)/F_baseline_, in which F_baseline_ was the mean calcium signal before the time zero.

### 2.5 EEG recording

After EEG recording cable and the laser stimulator was connected to the skulls of animals, mice were first habituated to a plexiglass cuboid container (16 cm in length, 120 cm in width, 100 cm in height) for 5 min. EEG signals were continuously recorded by the PowerLab 16/35 amplifier system (PL3516, AD Instruments, Dunedin, New Zealand) and LabChart Pro version 8.1.13 software (MLU60/8, AD Instruments, Dunedin, New Zealand). The raw EEG data were sampled at the frequency of 1000 Hz and were bandpass filtered with 0.3 Hz to 50 Hz for further analysis. For the burst‐suppression ratio (BSR) calculation, 1.0% isoflurane was inlet constantly for more than 30 min until the burst‐suppression wave appeared regularly. The 2‐min blue laser (473 nm, 20 Hz, 20 ms, 15 mW from tips, Thinker Tech, Nanjing, China) was delivered to activate neurons, whereas 2‐min yellow laser (594 nm, 1 Hz, 1 s, 10 mW from tips, Thinker Tech, Nanjing, China) was applied for inhibition. For spectral analysis, mice were exposed to 0.8% isoflurane anesthesia for about 30 min. After the EEG signals were stabilized, optical manipulation was applied as previously mentioned. Burst‐suppression ratio (BSR) and spectral analysis were accomplished by MATLAB (R2018a; MathWorks, Natick, MA, USA) and custom written MATLAB codes as previously reported.^21^ The frequency bands were composed of delta (δ: 0.3‐4 Hz), theta (θ: 4‐10 Hz), alpha (α: 10‐15 Hz), beta (β: 15‐25 Hz), and gamma(γ: 25‐50 Hz) waves.

### 2.6 Behavioral tests

Behavioral tests to examine the induction and emergence times were adopted from methods described before.^22^ Mice were freely placed in a horizontal transparent Plexiglas cylinder (45 cm in length, 12 cm in diameter) for 30 min for habituation. Then, anesthesia was induced by 1.4% isoflurane with 100% O_2_ at a flow rate of 1.5 L/min. The cylinder was rotated 180‐degree every 15 s until the mice behaved as loss of righting reflex (LORR) and could not turn itself prone onto all four limbs. The time from the initiation of isoflurane inhalation to LORR was considered as induction time. After 30 min of continuous anesthesia, the delivery of isoflurane was interrupted and the cylinder was rotated as described above to evaluate the time when mice could independently turn from the supine position with at least three paws reaching the bottom of the cylinder. The moment from the cessation of anesthesia to the recovery of righting reflex (RORR) was defined as emergence time. During the whole experiment, a heating pad was taped to the bottom of the cage so as to keep the body temperature of mice around 37.5°C.

### 2.7 Immunohistochemistry

Mice were deeply anesthetized with isoflurane and cardially perfused with 4% paraformaldehyde (PFA) followed by 0.9% saline. Brains were post‐fixed for 2 hours in 4% PFA at 4℃ temperature and then dehydrated by 30% sucrose in PBS serially. Brains were coronally sectioned at 40 μm using a cryostat microtome (CM1200, Leica). The brain sections were washed in phosphate‐buffered saline (PBS, pH = 7.4) and then blocked with 5% normal donkey serum in PBS with 0.3% Triton X‐100 for 2 hours at room temperature. Primary antibodies, including mouse anti‐c‐Fos (1:1000, Abcam) and rabbit anti‐Tph2 (1:200, Synaptic Systems), were incubated at 4°C overnight, respectively, followed by incubation of secondary antibodies, including donkey anti‐rabbit Alexa 488 (1:400, Jackson ImmunoResearch) or donkey anti‐mouse Cy3 (1:400, Jackson ImmunoResearch) for 2 hours at room temperature. And then, the brain slices were mounted in Fluoromount‐G (Millipore) and imaged by laser confocal microscopes (FV1200, Olympus).

### 2.8 Slice recording

After viral expression, acute coronal brain slices containing DRN were obtained at 300 um thickness in a vibratome (Leica VT1200S) while being submerged in an ice‐cold 95.0% O_2_/5.0% CO_2_ saturated solution containing 124 mM NaCl, 25 mM NaHCO_3_, 2.5 mM KCl, 1 mM NaH_2_PO_4_, 2 mM CaCl_2_, 2 mM MgSO_4_, and 37 mM glucose, and incubated for 60 min at 35℃ in 95.0% O2/5.0% CO2 saturated artificial cerebrospinal fluid (ACSF) containing 124 mM NaCl, 24 mM NaHCO_3_, 3.8 mM KCl, 1.2 mM NaH_2_PO_4_, 1 mM MgCl_2_, 2.5 mM CaCl_2_, and 10 mM glucose. In the recording chamber continuously perfused with oxygenated ACSF (1.5‐2 ml/min) at room temperature, whole‐cell recording was performed with micropipettes (1.5 mm outer diameter [OD], 1.1 mm inner diameter [ID]). The pipette inner solution contained 130 mM K‐gluconate, 4 mM KCl, 1 mM MgCl_2_, 10 mM HEPES, 0.3 mM EGTA, 4 mM Mg‐ATP, and 0.3 mM Na‐GTP (pH = 7.4). Current clamp was used to measure the electrophysiological effects of genetical manipulation of 5‐HT neurons in the DRN.

### 2.9 Statistical analysis

All statistical analyses were performed with Prism 8.0.1 (GraphPad, US) and MATLAB (MathWorks, US). Data were presented as mean ± SEM, and SEM was indicated by error bars in figures. Normality of data distributions was tested by the Shapiro‐Wilk tests. Two‐way ANOVA followed by Sidak's post hoc test was used to assess the statistical difference for chemogenetic experiments, one‐way ANOVA followed by Tukey's post hoc test for pharmacological experiments, and Student's *t* test for optogenetic and immunostaining experiments. Statistical significance levels are indicated as follows: *P < 0.05, **P < 0.01, and ***P < 0.001.

## 3 RESULTS

### 3.1 Neuronal activity of DRN 5‐HT neurons was inhibited during isoflurane anesthesia

**FIGURE 1:**
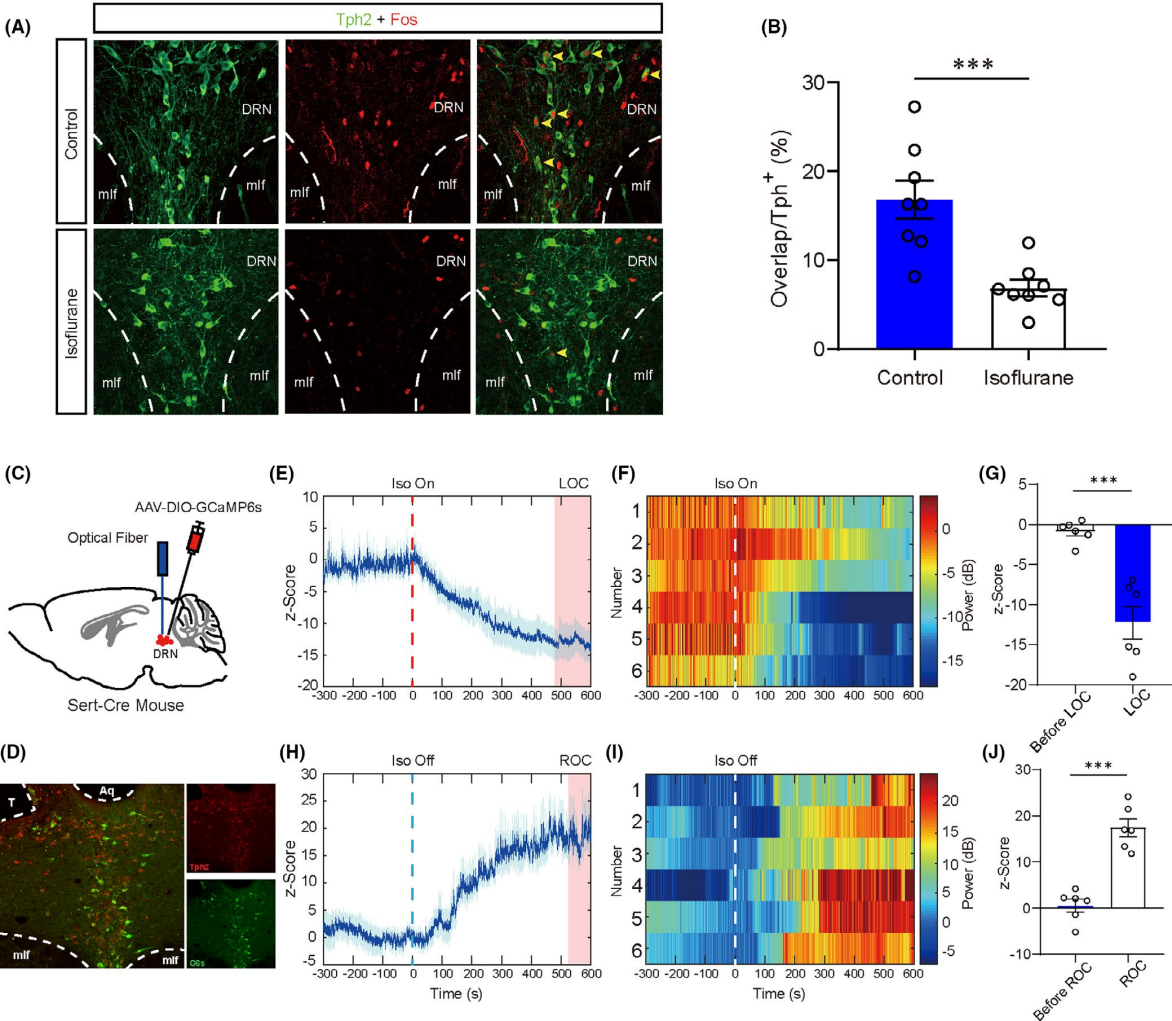
General anesthesia decreased the activities of 5‐HT neurons in the DRN. A, Representative immunofluorescent images of double staining of Tph2 and Fos in control and isoflurane anesthesia groups. B, Percentage of Fos‐positive 5‐HT neurons was significantly decreased in the isoflurane anesthesia group (6.883 ± 0.9123%), compared to the control group (16.82 ± 2.158%). C, Schematic illustration of virus injection. D, Verification of virus expression and track of optical fiber (red, anti‐Tph2; green, GCaMP6 s). E, Calcium signal decreased after loss of consciousness induced by isoflurane anesthesia (mean ± SEM, indicated by the full line and shaded area, and time to loss of consciousness indicated by the pink shaded areas). F, Heatmaps of calcium signal before and after initiation of isoflurane. G, Calcium signal was significantly reduced before and after loss of consciousness (−0.8843±0.5483 vs −12.28±2.059, *P* < 0.001). H, Calcium signal increases after recovery of consciousness from isoflurane anesthesia (mean ± SEM, indicated by the full line and shaded area, and time to recovery of consciousness indicated by the pink shaded areas). I, Heatmaps of calcium signal before and after cessation of isoflurane. J. Calcium signal was significantly increased before and after recovery of consciousness (0.5619 ± 1.375 vs 17.42 ± 1.917, *P* < 0.001) (*P < 0.05, **P < 0.01, and ***P < 0.001, unpaired *t* test)

To investigate the activity of DRN 5‐HT neurons, we first examined the expression of immediate early gene, *c*‐*fos*, which is commonly accepted as a proxy of neuronal activity.^23^ Wild‐type mice were anesthetized under 1.4% isoflurane vaporized by oxygen at a flow rate of 1.0 L/min and then sacrificed for immunostaining. Co‐staining of Fos and tryptophan hydroxylase (Tph2), a key enzyme for 5‐HT synthesis,^24^ showed that the percentage of Fos‐positive 5‐HT neurons in the DRN decreased after 2‐h isoflurane anesthesia (6.883 ± 0.9123%) in comparison with the control group (16.82 ± 2.158%), indicating that the 5‐HT neuronal activities reduced (Figure 1A,B, *P* < 0.001).

To further assess the in vivo activity of DRN 5‐HT neurons, we injected the AAV‐DIO‐GCaMP6 s into the DRN of Sert‐Cre mice which expressed Cre recombinase driven by the serotonin reuptake transporter promoter to achieve Cre‐dependent expression of calcium indicator in the 5‐HT neurons (Figure 1C,D). Calcium activity of DRN 5‐HT neurons gradually decreased after the initiation of isoflurane anesthesia at a concentration of 1.4% (Figure 1E‐G), and the activity recovered after termination of anesthetic inhalation before the mice regained their consciousness (Figure 1H‐J). Collectively, these results showed that DRN 5‐HT neurons were suppressed by the isoflurane anesthesia and gradually reactivated as the isoflurane anesthesia faded away, implying the involvement of DRN 5‐HT neurons in the regulation of general anesthesia.

### 3.2 Optogenetic manipulation of DRN 5‐HT neurons altered the burst‐suppression pattern of EEG during anesthesia maintenance

**FIGURE 2:**
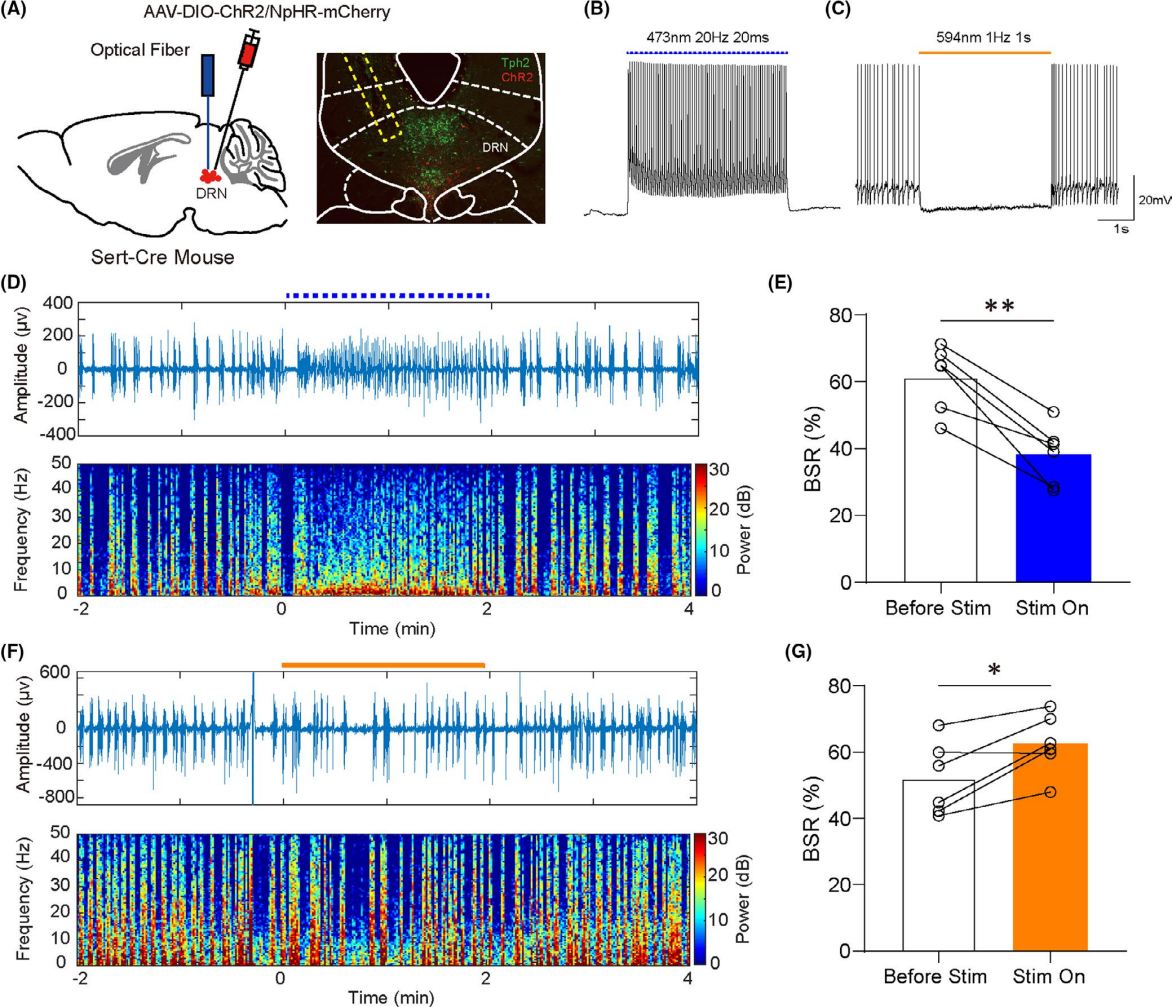
Optical stimulation of DRN 5‐HT neurons affected the depth of isoflurane anesthesia. A, Left, schematic illustration of vius injection. Right, verification of virus expression and track of optical fiber (red, ChR2‐mCherry; green: Tph2). B,C, Confirmation of ChR2 or NpHR virus efficiency. D, Representative peri‐stimulation raw EEG and EEG spectra of blue laser. E, Activation of DRN 5‐HT neurons significantly reduced the BSR during anesthesia maintenance (61.26 ± 4.016% vs 38.26 ± 3.638%, *P *= 0.0015). F, Representative peri‐stimulation raw EEG and EEG spectra of yellow laser. G, Inhibition of DRN 5‐HT neurons significantly increased the BSR during anesthesia maintenance (51.98 ± 4.513% vs 62.53 ± 3.686%, *P *= 0.0191) (*P < 0.05, **P < 0.01, and ***P < 0.001, paired *t* test)

To testify the regulatory effect of 5‐HT neurons in the DRN on the general anesthesia, the light‐sensitive channelrhodopsin (ChR2) or halorhodopsin (NpHR) was specifically expressed on the DRN 5‐HT neurons by injecting AAV‐DIO‐ChR2/NpHR‐mCherry virus into the DRN of Sert‐Cre mice (Figure 2A). Electrophysiological studies showed that 5‐HT neurons expressing ChR2 could effectively fire action potentials following 473 nm blue light pulses at the frequencies of 5 Hz, 10 Hz, and 20 Hz (Figure 2B, Figure S1), and those neurons expressing NpHR were strongly inhibited by continuously delivery of 594 nm yellow light (Figure 2C). During 1.0% isoflurane anesthesia maintenance, activation of DRN 5‐HT neurons by applying 20 Hz blue light pulses significantly reduced the burst‐suppression ratio (BSR) from 61.26 ± 4.016% to 38.26 ± 3.638% (Figure 2D,E, *P* = 0.0015). In contrast, optical inhibition of DRN 5‐HT neurons increased BSR (Figure 2F,G, 51.98 ± 4.513% vs 62.53 ± 3.686%, *P *= 0.0191). Overall, these changes of BSR suggested that DRN 5‐HT neurons could reduce the anesthetic depth during anesthesia maintenance.

### 3.3 Chemogenetic modulation of DRN 5‐HT neurons only affected the emergence time of isoflurane anesthesia

**FIGURE 3:**
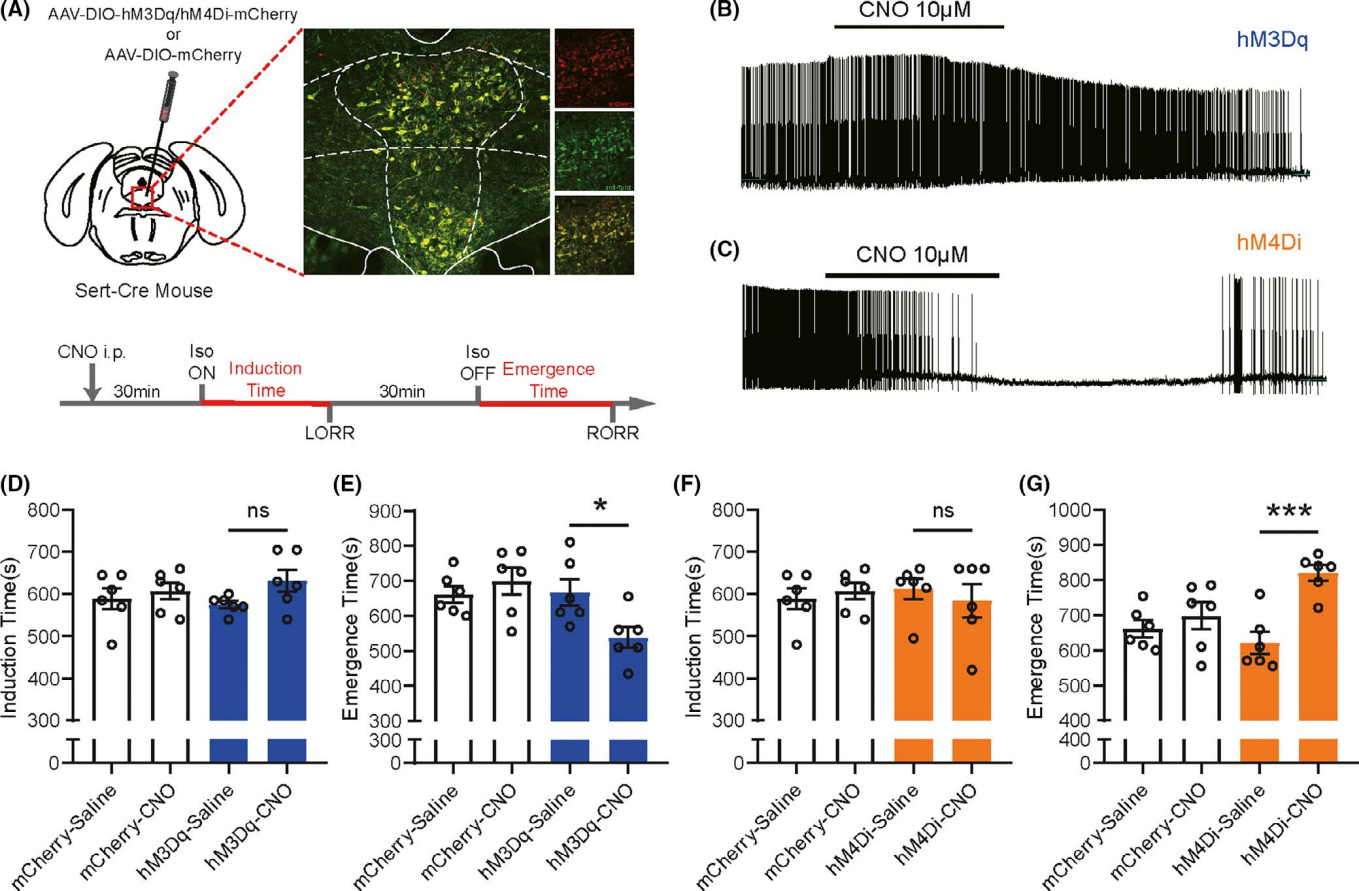
DRN 5‐HT neurons facilitated the emergence from isoflurane anesthesia. A, Top, schematic illustration of virus injection and verification of virus expression (red, hM3Dq/hM4Di‐mCherry; green: Tph2). Bottom, experimental protocol of assessment of time to LORR and RORR. B, C, Confirmation of hM3Dq or hM4Di virus efficiency. D, Activation of DRN 5‐HT neurons did not change the induction time (*P *= 0.6460). E, Activation of DRN 5‐HT neurons significantly shortened the emergence time (*P *= 0.0217). F, Inhibition of DRN 5‐HT neurons did not change the induction time (*P *= 0.4252). G, Inhibition of DRN 5‐HT neurons significantly prolonged the emergence time (*P *= 0.0135) (*P < 0.05, **P < 0.01, and ***P < 0.001, two‐way ANOVA followed by Sidak's post hoc test)

Behavioral tests were applied to further confirm the effect of DRN 5‐HT neurons on general anesthesia. By injecting AAV‐DIO‐hM3Dq/hM4Di‐mCherry viruses into the DRN of Sert‐Cre mice, two different designer receptors exclusively activated by designer drugs (DREADDs), hM3Dq or hM4Di, were expressed on the 5‐HT neurons (Figure 3A). Application of CNO onto the acute brain slices induced robust activation of hM3Dq‐expressing 5‐HT neurons and inhibition of hM4Di‐expressing 5‐HT neurons (Figure 3B,C). Vehicle or CNO was injected intraperitoneally 30 minutes before the initiation of 1.4% isoflurane to activate or inhibit DRN 5‐HT neurons, and conscious states of mice were measured by the righting reflex. Time to loss of righting reflex (LORR) after inhalation of isoflurane was considered as the induction time and time to recovery of righting reflex (RORR) as the emergence time. Although activation of DRN 5‐HT neurons showed little influence on the induction time (Figure 3D, *P*=0.6460), the emergence time was significantly shortened compared with control groups (Figure 3E, *P*=0.0217). On the contrary, inhibition of DRN 5‐HT neurons prolonged the emergence time (Figure 3G, *P*=0.0135), without affecting the induction time (Figure 3F, *P*=0.4252). Taken together, behavioral results demonstrate that DRN 5‐HT neurons could facilitate the emergence from general anesthesia.

### 3.4 Serotonergic neurons exerted their effect mainly through 5‐HT 1A and 2C receptors

**FIGURE 4:**
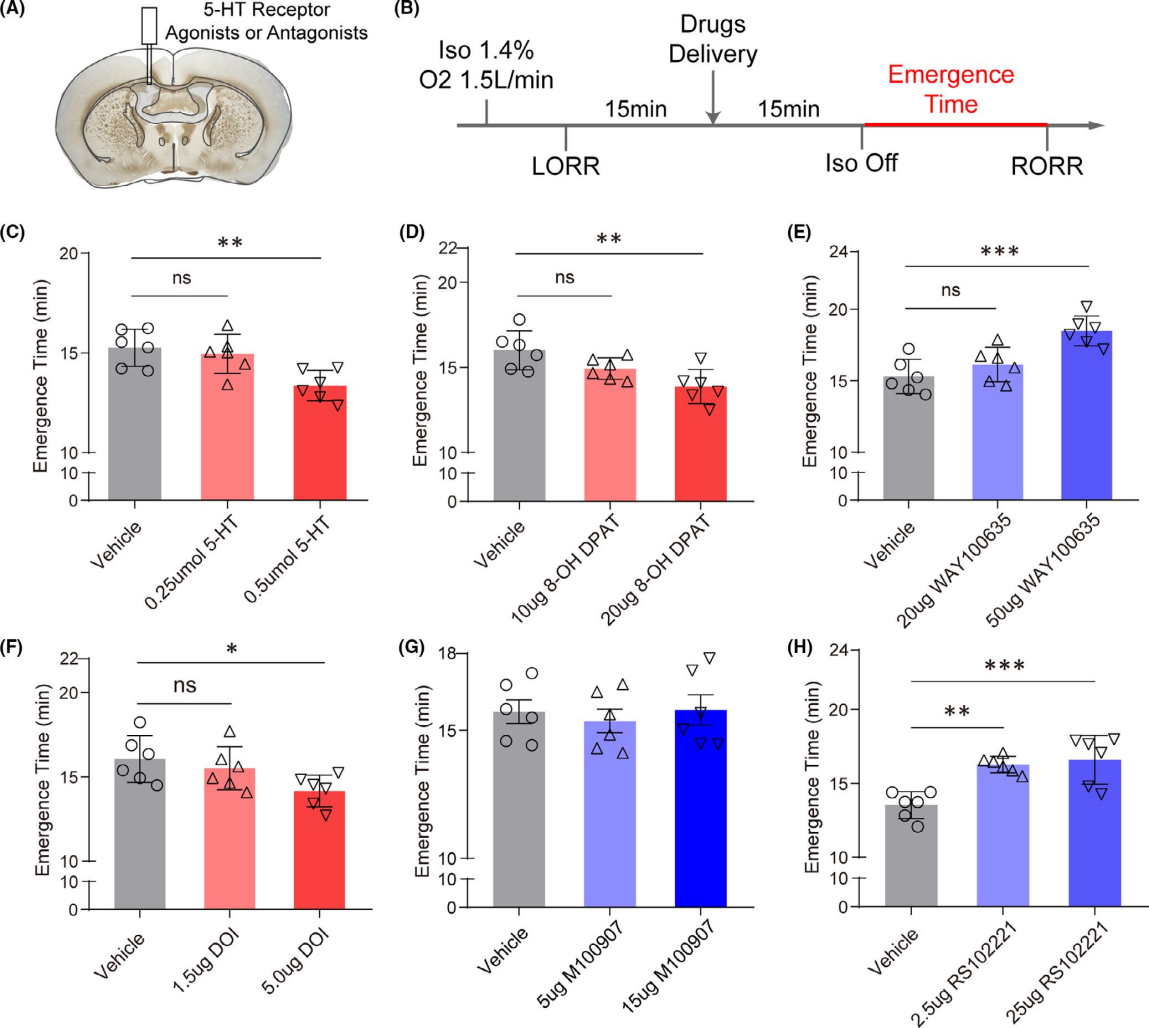
Intracerebroventricular infusion of 5‐HT and 5‐HT 1A or 2C receptor agonists or antagonists changed the emergence time of isoflurane anesthesia. A, Verification of cannula track for intracerebroventricular infusion. B, Experimental protocol of assessment of time to RORR. C, Intracerebroventricular application of 5‐HT shortened the emergence time in a dose‐dependent manner (*P *= 0.0049). D, Intracerebroventricular application of 5‐HT 1A receptor agonist shortened the emergence time in a dose‐dependent manner (*P *= 0.0053). E, Intracerebroventricular application of 5‐HT 1A receptor antagonist prolonged the emergence time in a dose‐dependent manner (*P* < 0.001). F, Intracerebroventricular application of 5‐HT 2A/2C receptor agonist shortened the emergence time in a dose‐dependent manner (*P *= 0.0470). G, Intracerebroventricular application of 5‐HT 2A receptor had no effect on the emergence time (*P *= 0.8154). H, Intracerebroventricular application of 5‐HT 2C receptor antagonist prolonged the emergence time in a dose‐dependent manner (*P* < 0.001) (*P < 0.05, **P < 0.01, and ***P < 0.001, one‐way ANOVA followed by Tukey's post hoc test)

Above results have shown that DRN 5‐HT neurons could facilitate emergence from general anesthesia, but several studies hinted the possibility that 5‐HT neurons might exert their functions by co‐releasing other neurotransmitters, like glutamate or gamma‐aminobutyric acid (GABA).^24^, ^25^ We then performed the pharmacological experiments on the SD rats to investigate whether the effect of activating DRN 5‐HT neurons was associated with the release of serotonin. By implantation of cannula above the lateral cerebral ventricle, 5‐HT was delivered by intracerebroventricular injection 15 minutes before cessation of isoflurane (Figure 4A,B). Although 0.25 umol 5‐HT microinjection did not affect the emergence time (*P*=0.7788), rats took significantly shorter time to regain the righting reflex with 0.5 umol 5‐HT injected intracerebroventricularly (*P*=0.0043), mimicking the effect of activating DRN 5‐HT neurons (Figure 4C). These results indicate that DRN 5‐HT neurons facilitate emergence from general anesthesia partially through serotonin signaling.

Serotonin could bind to diverse receptors including both ionotropic and metabotropic receptors, classified into 5‐HT 1‐7 receptors, which endowed the serotonergic systems with complex functions.^26^ To explore which receptors were involved in the emergence promoting effect of DRN 5‐HT neurons, agonists and antagonists of various serotonin receptors which have been implicated in the regulation of sleep and wakefulness were used to measure the behavioral effect on anesthesia.^12^ Intracerebroventricular application of the selective 5‐HT 1A receptor agonist, 8‐OH DPAT, significantly shortened the emergence time (*P*=0.0027, Figure 4D), whereas WAY‐100635, a selective 5‐HT 1A receptor antagonist prolonged the time to RORR (*P*=0.0005, Figure 4E), implying the involvement of 5‐HT 1A receptor in emergence. Moreover, intracerebroventricular infusion of 5‐HT 2A/2C receptor agonist, DOI, also accelerated the emergence from isoflurane anesthesia (*P*=0.0321, Figure 4F), and infusion of high selective 5‐HT 2C receptor antagonist (RS‐102221, *P*=0.006, Figure 4H) but not 5‐HT 2A receptor antagonist (M100907, *P*=0.8154, Figure 4G) achieved the opposite effect, suggesting the importance of 5‐HT 2C receptor. However, agonists or antagonists of other 5‐HT receptors, including 5‐HT 1B, 3, 6, and 7 receptors, showed little regulatory effect on emergence time of anesthesia (Figure S2). Overall, these results highlighted the involvement of 5‐HT 1A and 2C receptor signaling in the modulation of isoflurane anesthesia.

## 4 DISCUSSION

In this study, we combined the immunostaining with fiber photometry of calcium indicator to illustrate the reduction of DRN 5‐HT neuronal activity during general anesthesia. Then, through optogenetic and chemogenetic methods, we found that activation of DRN 5‐HT neurons could reduce the depth of anesthesia and facilitate emergence from isoflurane anesthesia. Finally, intracerebroventricular infusion of serotonin and agonists or antagonists of 5‐HT receptors implied the involvement of serotonin and 5‐HT 1A and 2C receptor signaling in the regulative effect of DRN 5‐HT neurons on general anesthesia.

Several studies reported that concentration of serotonin was decreased in the prefrontal cortex and hypothalamus during general anesthesia.^13^, ^14^ Indeed, we found that activity of DRN 5‐HT neurons was significantly decreased during isoflurane anesthesia, which was consistent with the idea that brainstem activating systems were inhibited during anesthetic‐induced consciousness.^2^ Previous studies using multi‐channel recording found that putative DRN 5‐HT neuronal activity was correlated to the behavioral arousal levels and significantly decreased during slow wave sleep (SWS).^27^, ^28^ Recently, fiber photometry of genetically identified 5‐HT neurons yielded the similar results that DRN 5‐HT neurons increased their activity when the mice transit from NREM sleep to wakefulness,^19^ reminiscent of the increase of calcium activity at emergence from anesthesia in our studies. Volatile anesthetics, including isoflurane, act through a wide array of molecular targets, in which the facilitation of GABA_A_ receptor plays a major role.^2^ DRN 5‐HT neurons have been shown to express GABA_A_ receptors.^29^ The reduction of neuronal activity during general anesthesia might be attributed to the enhanced inhibitory inputs from local interneurons or forebrain nuclei such as preoptic area (POA) through facilitation of GABA_A_ receptor signaling.^30^ As showed in the calcium imaging, the rapid recovery of DRN 5‐HT neuronal activity occurred in ahead of mice recovering consciousness after the cessation of isoflurane inhalation, indicating the potential involvement of DRN 5‐HT neurons in the emergence from anesthesia. Indeed, activation of DRN 5‐HT accelerated recovery to consciousness, and inhibition delayed the emergence period. These results further extended our findings that wake‐promoting neuropeptide, orexin, could activate DRN 5‐HT neurons to facilitate the emergence from general anesthesia.^20^

Based on in vitro slice recordings, we chose 20 Hz as the optimal photostimulation pattern for activating DRN 5‐HT neurons. However, it has been reported that DRN 5‐HT neurons could be fired at a broad range of stimulating frequency to accomplish their complex functions, from 5 Hz during quiet waking states to 20–30 Hz while actively waking, rewarding or punishments.^25^, ^31^, ^32^ Interestingly, optogenetic activation of DRN 5‐HT neurons by 25 Hz pulse induced a sudden increase of wakefulness probability at the expense of NREM and rapid eye movement (REM) sleep, but stimulation of DRN 5‐HT neurons at 3 Hz tended to generate sleep pressures and gradually led to NREM sleep.^19^ These studies combined with our finding in general anesthesia implied a bidirectional regulative effects of DRN 5‐HT neurons and emphasized the importance for further studies to measure the firing pattern transitions of these neurons during wakefulness and sleep or anesthesia, using methods of higher resolution, like the multi‐channel recording of optogenetic defined cell types, or the calcium photometry of single neurons.^33^, ^34^

Serotonin could bind to diverse receptors of distinct characteristics which endowed the serotonergic system with the ability to mediate a wide array of behavioral functions.^24^ The 5‐HT 1A receptors are the metabotropic receptors which could hyperpolarize the membrane potential through activation of G‐protein‐coupled potassium channel.^26^ Previous studies have demonstrated that systematic application of 5‐HT 1A receptor agonists, flesinoxan or 8‐OH DPAT, increased the wakefulness and reduced the slow wave sleep (SWS) and REM sleep.^35^ This wake‐promoting effect was likely produced by the inhibition of sleep active neurons in the POA and facilitation of the wake‐associated neurotransmitters release, including acetylcholine (ACh) and noradrenaline (NA),^12^, ^36^ which might also underline the mechanism of facilitating emergence from general anesthesia. On the other hand, it has been reported that subcutaneous administration of 5‐HT 2A/2C receptor agonists, DOI, prolonged the wakefulness, but shortened SWS and REM sleep; however, this effect was proven to be mediated by the 5‐HT 2A receptors.^37^ Here, we demonstrated that antagonist of 5‐HT 2C receptors but not 5‐HT 2A receptors generated the opposite effect of DOI, which indicates a probably different mechanism of serotonergic system in the regulation of general anesthesia against sleep. Nevertheless, further studies should be performed to interpret this difference in the future.

There are also some limitations of the present study. Although 5‐HT 1A and 2C receptors were identified as possible mechanisms of serotonergic facilitation of emergence from anesthesia, the two receptors exerted opposite excitatory and inhibitory effect on the downstream targets. DRN 5‐HT neurons innervated the majority of brain regions, previous studies demonstrated that there were subgroups of 5‐HT neurons in the DRN with distinct projection regions. DRN 5‐HT neurons projecting to orbitofrontal cortex (OFC) tended to have compensatory innervations with those projecting to central amygdala (CeA).^16^ The divergent innervating properties and various levels of downstream nuclei expressions of subtypes of 5‐HT receptors implied possible mechanism of two receptors with opposite characteristics to exert similar effects. Besides, intracerebroventricular infusion of 5‐HT indicated the involvement of serotoninergic mechanism in the emergence promoting effect of DRN 5‐HT neurons, but the probability of co‐releasing of other neurotransmitters such as glutamate when DRN 5‐HT neurons were activated could not be excluded, and this possibility should also be tested in the future.

## 5 CONCLUSION

In conclusion, the present studies provided several evidences for the role of DRN 5‐HT neurons to facilitate emergence from isoflurane anesthesia, and this effect was at least partially mediated by the 5‐HT 1A and 2C receptors. This study further expanded the brainstem ascending systems affecting general anesthesia, and because serotonergic systems are highly associated with emotional behaviors, our findings may give some hints for the perioperative psychiatric disorders and anesthesia management of patients with neuropsychiatric diseases or under treatments targeting serotonergic systems.

## CONFLICT OF INTEREST

The authors state that there are no conflicts of interest to disclose.

## Supporting information

Figure S1

Figure S2

## Data Availability

The data that support the findings of the present study are available from the corresponding authors upon reasonable request.

## References

[1] Brown EN , Purdon PL , Van Dort CJ . General anesthesia and altered states of arousal: a systems neuroscience analysis. Ann Rev Neurosci. 2011;34:601‐628. 21513454 10.1146/annurev-neuro-060909-153200 PMC3390788

[2] Franks NP . General anaesthesia: from molecular targets to neuronal pathways of sleep and arousal. Nat Rev Neurosci. 2008;9(5):370‐386. 18425091 10.1038/nrn2372

[3] Makaryus R , Lee H , Yu M , et al. The metabolomic profile during isoflurane anesthesia differs from propofol anesthesia in the live rodent brain. J Cereb Blood Flow Metab. 2011;31(6):1432‐1442. 21266982 10.1038/jcbfm.2011.1 PMC3130322

[4] Xie H , Chung DY , Kura S , et al. Differential effects of anesthetics on resting state functional connectivity in the mouse. J Cereb Blood Flow Metab. 2020;40(4):875‐884. 31092086 10.1177/0271678X19847123 PMC7168791

[5] Brown EN , Lydic R , Schiff ND . General anesthesia, sleep, and coma. N Engl J Med. 2010;363(27):2638‐2650. 21190458 10.1056/NEJMra0808281 PMC3162622

[6] Franks NP , Zecharia AY . Sleep and general anesthesia. Can J Anaesth. 2011;58(2):139‐148. 21170623 10.1007/s12630-010-9420-3

[7] Dong HL , Fukuda S , Murata E , Zhu Z , Higuchi T . Orexins increase cortical acetylcholine release and electroencephalographic activation through orexin‐1 receptor in the rat basal forebrain during isoflurane anesthesia. Anesthesiology. 2006;104(5):1023‐1032. 16645455 10.1097/00000542-200605000-00019

[8] Kelz MB , Sun Y , Chen J , et al. An essential role for orexins in emergence from general anesthesia. Proc Natl Acad Sci USA. 2008;105(4):1309‐1314. 18195361 10.1073/pnas.0707146105 PMC2234134

[9] Taylor NE , Van Dort CJ , Kenny JD , et al. Optogenetic activation of dopamine neurons in the ventral tegmental area induces reanimation from general anesthesia. Proc Natl Acad Sci USA. 2016;113(45):12826‐12831. 27791160 10.1073/pnas.1614340113 PMC5111696

[10] Zhang Z , Ferretti V , Guntan I , et al. Neuronal ensembles sufficient for recovery sleep and the sedative actions of alpha2 adrenergic agonists. Nat Neurosci. 2015;18(4):553‐561. 25706476 10.1038/nn.3957 PMC4836567

[11] Liu C , Zhou X , Zhu Q , et al. Dopamine neurons in the ventral periaqueductal gray modulate isoflurane anesthesia in rats. CNS Neurosci Ther. 2020;26(11):1121‐1133. 32881314 10.1111/cns.13447 PMC7564192

[12] Monti JM . Serotonin control of sleep‐wake behavior. Sleep Med Rev. 2011;15(4):269‐281. 21459634 10.1016/j.smrv.2010.11.003

[13] Mukaida K , Shichino T , Koyanagi S , Himukashi S , Fukuda K . Activity of the serotonergic system during isoflurane anesthesia. Anesth Analg. 2007;104(4):836‐839. 17377090 10.1213/01.ane.0000255200.42574.22

[14] Roizen MF , White PF , Eger EI 2nd , Brownstein M . Effects of ablation of serotonin or norepinephrine brain‐stem areas on halothane and cyclopropane MACs in rats. Anesthesiology. 1978;49(4):252‐255. 697079 10.1097/00000542-197810000-00005

[15] Monti JM . The structure of the dorsal raphe nucleus and its relevance to the regulation of sleep and wakefulness. Sleep Med Rev. 2010;14(5):307‐317. 20153669 10.1016/j.smrv.2009.11.004

[16] Ren J , Friedmann D , Xiong J , et al. Anatomically defined and functionally distinct dorsal raphe serotonin sub‐systems. Cell. 2018;175(2):472‐487 e420. 30146164 10.1016/j.cell.2018.07.043 PMC6173627

[17] Mohr AA , Garcia‐Serrano AM , Vieira JP , Skoug C , Davidsson H , Duarte JM . A glucose‐stimulated BOLD fMRI study of hypothalamic dysfunction in mice fed a high‐fat and high‐sucrose diet. J Cereb Blood Flow Metab. 2020:271678X20942397. 10.1177/0271678X20942397 PMC8217889 32757742

[18] Ito H , Yanase M , Yamashita A , et al. Analysis of sleep disorders under pain using an optogenetic tool: possible involvement of the activation of dorsal raphe nucleus‐serotonergic neurons. Mol Brain. 2013;6:59. 24370235 10.1186/1756-6606-6-59 PMC3879646

[19] Oikonomou G , Altermatt M , Zhang R‐W , et al. The serotonergic raphe promote sleep in zebrafish and mice. Neuron. 2019;103(4):686‐701 e688. 31248729 10.1016/j.neuron.2019.05.038 PMC6706304

[20] Yang C , Zhang L , Hao H , Ran M , Li J , Dong H . Serotonergic neurons in the dorsal raphe nucleus mediate the arousal‐promoting effect of orexin during isoflurane anesthesia in male rats. Neuropeptides. 2019;75:25‐33. 30935682 10.1016/j.npep.2019.03.004

[21] Li J , Li H , Wang D , et al. Orexin activated emergence from isoflurane anaesthesia involves excitation of ventral tegmental area dopaminergic neurones in rats. Br J Anaesth. 2019;123(4):497‐505. 31399212 10.1016/j.bja.2019.07.005

[22] Zhang L‐N , Yang C , Ouyang P‐R , et al. Orexin‐A facilitates emergence of the rat from isoflurane anesthesia via mediation of the basal forebrain. Neuropeptides. 2016;58:7‐14. 26919917 10.1016/j.npep.2016.02.003

[23] Guzowski JF , Timlin JA , Roysam B , McNaughton BL , Worley PF , Barnes CA . Mapping behaviorally relevant neural circuits with immediate‐early gene expression. Curr Opin Neurobiol. 2005;15(5):599‐606. 16150584 10.1016/j.conb.2005.08.018

[24] Okaty BW , Commons KG , Dymecki SM . Embracing diversity in the 5‐HT neuronal system. Nat Rev Neurosci. 2019;20(7):397‐424. 30948838 10.1038/s41583-019-0151-3

[25] Liu Z , Zhou J , Li YI , et al. Dorsal raphe neurons signal reward through 5‐HT and glutamate. Neuron. 2014;81(6):1360‐1374. 24656254 10.1016/j.neuron.2014.02.010 PMC4411946

[26] Filip M , Bader M . Overview on 5‐HT receptors and their role in physiology and pathology of the central nervous system. Pharmacol Rep. 2009;61(5):761‐777. 19903999 10.1016/s1734-1140(09)70132-x

[27] Kirby LG , Pernar L , Valentino RJ , Beck SG . Distinguishing characteristics of serotonin and non‐serotonin‐containing cells in the dorsal raphe nucleus: electrophysiological and immunohistochemical studies. Neuroscience. 2003;116(3):669‐683. 12573710 10.1016/s0306-4522(02)00584-5 PMC2832757

[28] Trulson ME , Jacobs BL . Raphe unit activity in freely moving cats: correlation with level of behavioral arousal. Brain Res. 1979;163(1):135‐150. 218676 10.1016/0006-8993(79)90157-4

[29] Ren J , Isakova A , Friedmann D , et al. Single‐cell transcriptomes and whole‐brain projections of serotonin neurons in the mouse dorsal and median raphe nuclei. Elife. 2019;8:10.7554/eLife.49424 PMC6812963 31647409

[30] Moore J , Chen J , Han BO , et al. Direct activation of sleep‐promoting VLPO neurons by volatile anesthetics contributes to anesthetic hypnosis. Curr Biol. 2012;22(21):2008‐2016. 23103189 10.1016/j.cub.2012.08.042 PMC3628836

[31] Cohen JY , Amoroso MW , Uchida N . Serotonergic neurons signal reward and punishment on multiple timescales. Elife. 2015;4:10.7554/eLife.06346 PMC4389268 25714923

[32] McGinty DJ , Harper RM . Dorsal raphe neurons: depression of firing during sleep in cats. Brain Res. 1976;101(3):569‐575. 1244990 10.1016/0006-8993(76)90480-7

[33] Anikeeva P , Andalman AS , Witten I , et al. Optetrode: a multichannel readout for optogenetic control in freely moving mice. Nat Neurosci. 2011;15(1):163‐170. 22138641 10.1038/nn.2992 PMC4164695

[34] Zong W , Wu R , Li M , et al. Fast high‐resolution miniature two‐photon microscopy for brain imaging in freely behaving mice. Nat Methods. 2017;14(7):713‐719. 28553965 10.1038/nmeth.4305

[35] Monti JM , Jantos H . Dose‐dependent effects of the 5‐HT1A receptor agonist 8‐OH‐DPAT on sleep and wakefulness in the rat. J Sleep Res. 1992;1(3):169‐175. 10607047 10.1111/j.1365-2869.1992.tb00033.x

[36] Fink KB , Göthert M . 5‐HT receptor regulation of neurotransmitter release. Pharmacol Rev. 2007;59(4):360‐417. 18160701 10.1124/pr.107.07103

[37] Monti JM , Jantos H . Effects of the serotonin 5‐HT2A/2C receptor agonist DOI and of the selective 5‐HT2A or 5‐HT2C receptor antagonists EMD 281014 and SB‐243213, respectively, on sleep and waking in the rat. Eur J Pharmacol. 2006;553(1‐3):163‐170. 17059817 10.1016/j.ejphar.2006.09.027

