# Supplementary figures and images for: Dorsal raphe serotonergic neurons promote arousal from isoflurane anesthesia
Source: CNS Neurosci Ther. 2021 May 11;27(8):941–50. doi: 10.1111/cns.13656 (PMC8265942; doi:10.1111/cns.13656)

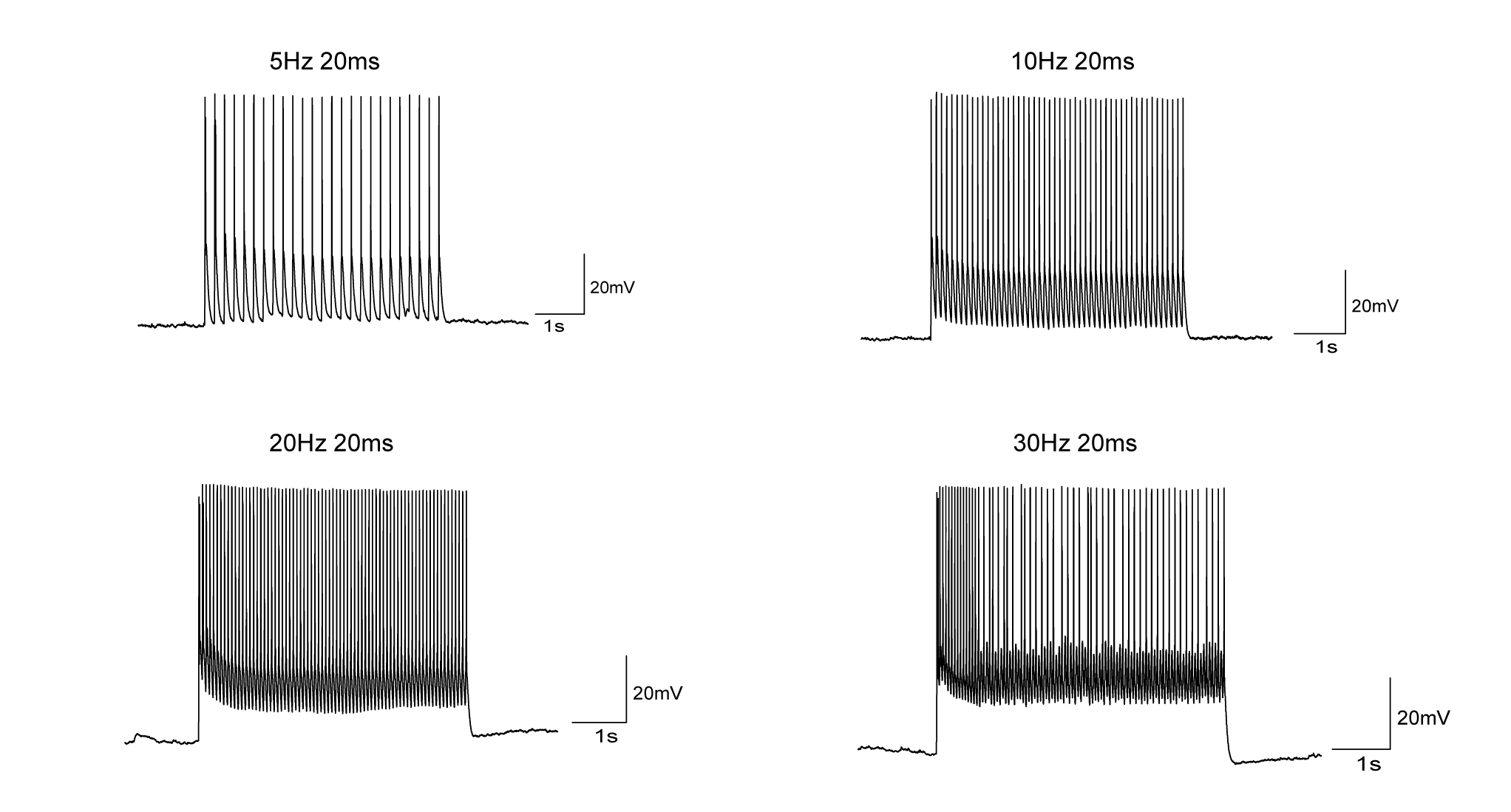

Supplement: Supplementary file 1 — Figure S1 [file CNS-27-941-s001.tif]

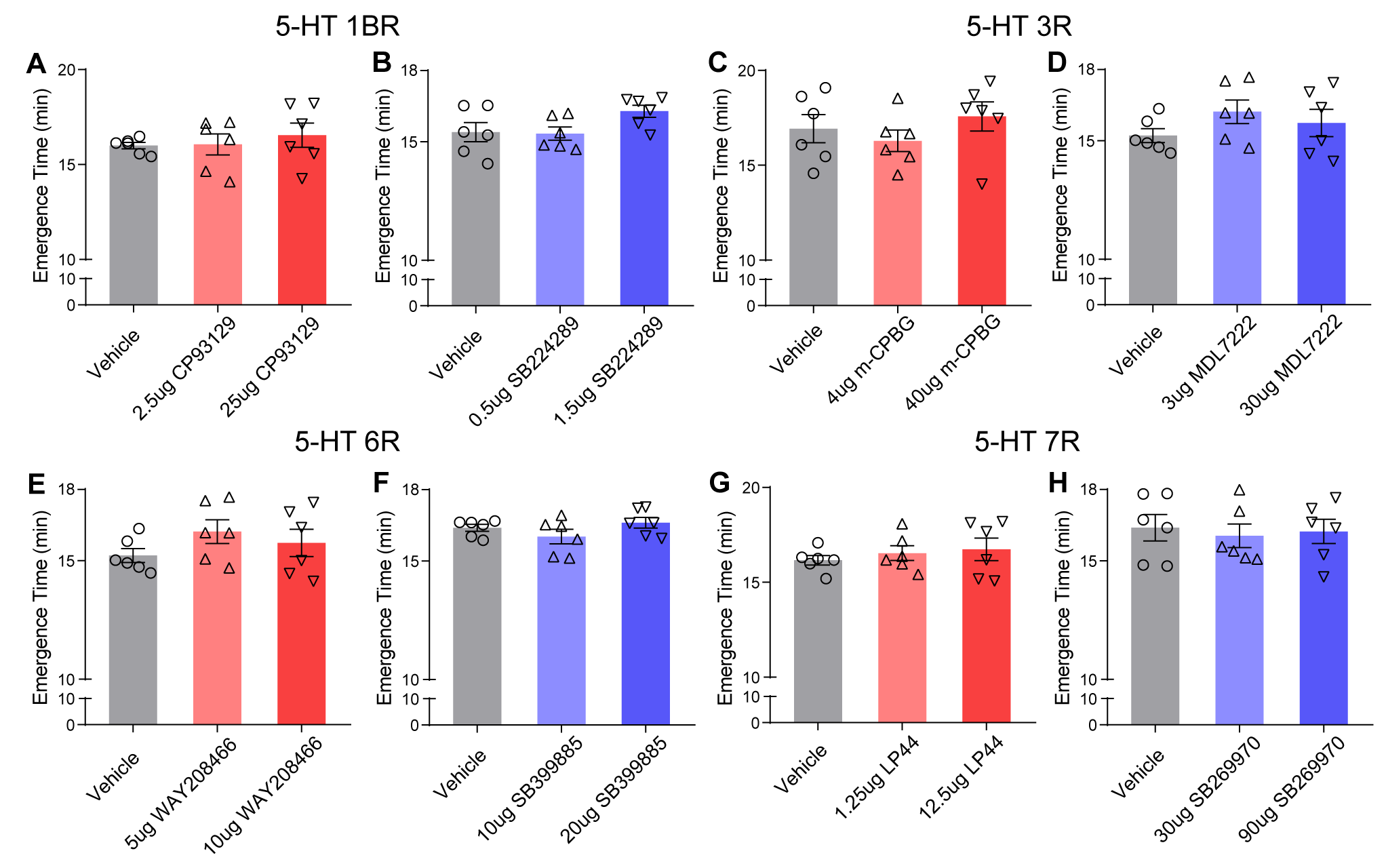

Supplement: Supplementary file 2 — Figure S2 [file CNS-27-941-s002.tif]
